# Effect of Grain Size on Polycrystalline Copper Finish Quality of Ultra-Precision Cutting

**DOI:** 10.3390/mi16101133

**Published:** 2025-09-30

**Authors:** Chuandong Zhang, Xinlei Yue, Kaiyuan You, Wei Wang

**Affiliations:** 1 School of Mechanical and Electrical Engineering, University of Electronic Science and Technology of China, Chengdu 611731, China; zcd@uestc.edu.cn (C.Z.); yuexinlei2024@163.com (X.Y.); 2 Yangtze Delta Region Institute (Quzhou), University of Electronic Science and Technology of China, Quzhou 324003, China

**Keywords:** polycrystalline copper, ultra-precision cutting, grain size, surface integrity

## Abstract

Polycrystalline copper optics are widely utilized in infrared systems due to their exceptional electrical and thermal conductivity combined with favorable machining characteristics. The grain size profoundly influences both surface quality consistency and fundamental material removal behavior during processing. This investigation employs multiscale numerical modeling to simulate nanoscale cutting processes in polycrystalline copper with controlled grain structures, coupled with experimental ultra-precision machining validation. Comprehensive analysis of stress distribution, subsurface damage formation, and cutting force evolution reveals that refined grain structures promote more homogeneous plastic deformation, resulting in superior surface finish with reduced roughness and diminished grain boundary step formation. However, the enhanced grain boundary density in fine-grained specimens necessitates increased cutting energy input. These findings establish critical process–structure–property relationships essential for advancing precision manufacturing of copper-based optical systems.

## 1. Introduction

Polycrystalline copper holds a pivotal position in optical applications as a critical functional material. This material exhibits exceptional electrical and thermal conductivity alongside balanced mechanical properties, making it the predominant industrial form of copper. In contrast to the highly ordered single-crystal structure of monocrystalline copper, the intrinsic presence of grain boundaries constitutes the defining feature of polycrystalline copper, profoundly governing its macroscopic behavior. The grain dimensions directly dictate the material removal process, consequently governing the quality of the machined surface. Precise optimization of grain size distribution and boundary configurations becomes fundamentally essential for achieving optical-grade surface finishes in advanced precision systems.

In the field of optical component manufacturing, the selection of appropriate workpiece processing methods plays an irreplaceable role in enhancing optical performance and simplifying light path configurations. Numerous advanced machining methods have been developed and implemented, including emerging hybrid technologies such as ultra-precision grinding, magnetorheological finishing, and femtosecond laser machining. For polycrystalline copper materials, the predominant approach is ultra-precision single-point diamond turning (SPDT). This technique leverages the nanoscale edge acuity and exceptional hardness of natural single-crystal diamond tools to achieve optical-quality surface fabrication.

Grain size serves as a critical factor influencing the ultra-precision machining quality of polycrystalline materials. In the realm of ultra-precision machining, variations in grain size of polycrystalline copper profoundly affect material removal mechanisms and surface integrity. Coarse-grained structures are prone to dislocation pile-up and significant stress concentration during machining, resulting in surface grain boundary steps that significantly compromise surface finish. Conversely, The Hall–Petch effect in fine-grained structures imparts higher strength and necessitates greater cutting forces to overcome the impediment to dislocation slip posed by abundant grain boundaries, resulting in significant cutting force oscillations. Nevertheless, this refinement process produces a more polished surface. Cao H et al. [1] investigated the influence of grain size on the deformation mechanisms and cutting performance of polycrystalline γ-TiAl alloy during nano-cutting through molecular dynamics simulations. It was revealed that the dominant plastic deformation mechanism transitions from dislocation slip to grain boundary slip. Furthermore, grain refinement was found to induce increased subsurface defects, a reduction in average cutting force, and gradient variations in stress distribution within the cutting zone. Li R et al. [2] demonstrated that the elastic modulus and lattice phase transitions of polycrystalline AlN show strong dependence on both average grain size and loading temperature, with larger grain sizes correlating with higher elastic moduli. Xue Z et al. [3] employed molecular dynamics simulations to examine nanoscale cutting mechanisms in polycrystalline tin, complemented by single-point diamond turning experiments and microscratch tests to elucidate material removal and subsurface deformation mechanisms.

The underlying mechanisms governing grain size effects on nanoscale material removal processes remain insufficiently understood, necessitating further systematic investigation. Molecular dynamics (MD) simulation has emerged as a powerful methodology for elucidating nanoscale cutting mechanisms, enabling comprehensive analysis of cutting forces, stress distributions, and temperature fields while simultaneously capturing atomic-scale phenomena including phase transformations and crystalline defect evolution. Through MD simulations, researchers have successfully unraveled ultra-precision process. Yao et al. [4] employed CGMD simulations to analyze the impact of carbon binder domain (CBD) parameters on electrode slurry properties and dried microstructure. Results indicate that CBD size and density dominate slurry viscosity and density, while active material (AM) particle distribution shows negligible influence. Reducing CBD size enhances particle agglomeration during drying. A CBD size of 6 μm or larger is proposed to minimize agglomeration and improve electrode performance. This study offers key insights for optimizing electrode slurry formulation and drying processes. Liu et al. [5] investigated residual stress distributions along different crystallographic orientations during rotational grinding of monocrystalline silicon wafers, employing molecular dynamics simulations coupled with analysis of actual abrasive cutting directions. Their findings demonstrated that the effective cutting direction of abrasives varied continuously from the wafer center to the edge due to curved grinding trajectories, and residual stress mapping showed maximum stress locations deviating from the [1 1 0] crystallographic orientation as a function of cutting direction variations. Papanikolaou et al. [6] investigated the influence of grain size on the cutting performance of polycrystalline aluminum during nano-cutting via molecular dynamics simulations. The results demonstrated that reducing grain size leads to a decrease in cutting forces and friction coefficient due to grain boundary sliding and thermal softening of the material. Meanwhile, fine-grained structures elevate the temperature in the cutting zone by reducing thermal conductivity. For the first time, it was observed that finer grains near the cutting region can relieve grain boundary residual stresses, a phenomenon analogous to the heat treatment effect.

This study systematically investigates subsurface damage evolution and surface integrity characteristics in polycrystalline copper with varying grain sizes during machining processes. By employing molecular dynamics simulation techniques and performing ultra-precision machining experiments, the research elucidates the machining behavior of polycrystalline copper across different grain structures, with particular emphasis on dislocation nucleation and propagation dynamics, stress distribution patterns, and cutting force fluctuations. The work provides fundamental insights into surface formation mechanisms during precision machining of polycrystalline copper with controlled grain size distributions.

## 2. MD Simulation

### 2.1. Model Setup Configuration

**Figure 1:**
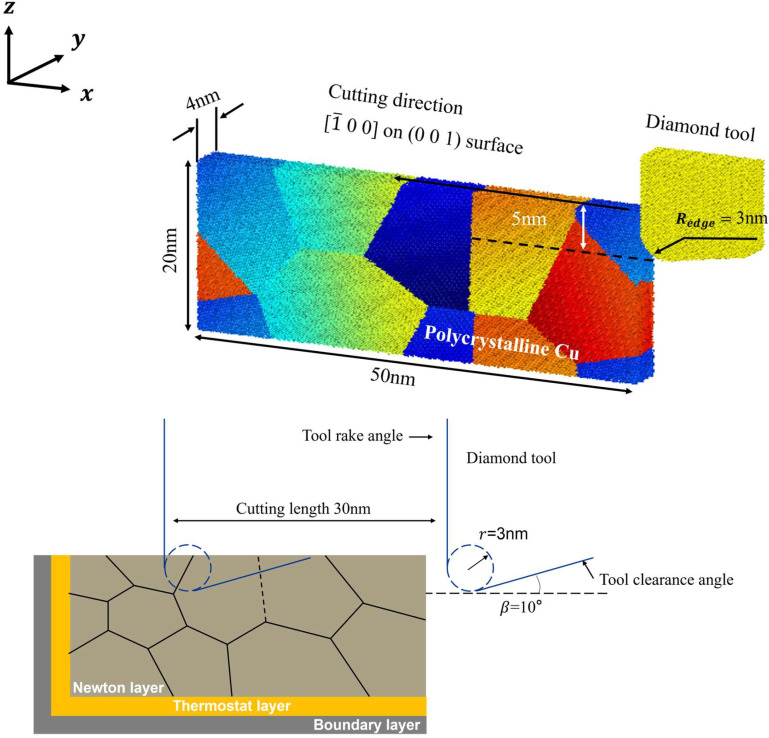
Polycrystalline copper model.

To investigate nanoscale damage evolution during cutting of polycrystalline materials, a molecular dynamics model of nanoscale cutting was established using the Large-scale Atomic/Molecular Massively Parallel Simulator (LAMMPS) coupled with the polycrystalline structure modeling software Atomsk (version beata 0.13.1) [7]. As illustrated in the accompanying Figure 1 and Table 1, polycrystalline copper models with four different grain sizes were constructed in this study using the Voronoi algorithm implemented in the Atomsk software, with overall dimensions of 500 × 40 × 200 ${nm}^{3}$, where individual grains are color-coded and randomly oriented. The model is broadly divided into three layers: the Newtonian layer, the thermostat layer, and the fixed boundary layer. The boundary layer is used to secure the workpiece, while the atoms in the Newtonian layer adhere to Newton’s laws of motion. The diamond cutting tool was configured with a 0° rake angle, 10° clearance angle, and 3 nm edge radius, executing the cutting process at 5 nm depth of cut and 0.1 nm/fs cutting velocity along the x-direction. Periodic boundary conditions were applied in the X, Y, and Z directions of the model. Prior to the cutting simulation, the conjugate gradient method was employed to minimize the energy of the system, thereby eliminating initial unstable configurations. The system was then fully relaxed at 300 K to achieve an equilibrium state.

The selection of the potential function critically influences the rationality and accuracy of the simulation during the cutting process. The Embedded Atom Method (EAM), widely used for metals, was adopted to describe the interatomic interactions within the polycrystalline copper workpiece. For the diamond indenter, which involves covalent interactions, the Tersoff potential was employed to characterize the atomic forces between its atoms. At the interface between the diamond indenter and the copper workpiece, where non-metal and metal atoms interact, the Morse potential is typically applied; accordingly, this study utilized the Morse potential to describe the cross-interaction between carbon (C) and copper (Cu) atoms. The molecular dynamics (MD) simulation was conducted under the NVT.

### 2.2. Results and Analysis

#### 2.2.1. Stress Field Analysis

To elucidate material removal mechanisms in polycrystalline copper, the shear and hydrostatic stress distributions within the machining zone were analyzed to characterize stress states and morphological evolution in both the workpiece and chip, with visualization performed using Ovito (basic – 3.12.2) [8]. The variation in grain size significantly alters stress field characteristics during cutting due to differences in grain boundary density and individual grain dimensions traversed by the tool [9].

**Figure 2:**
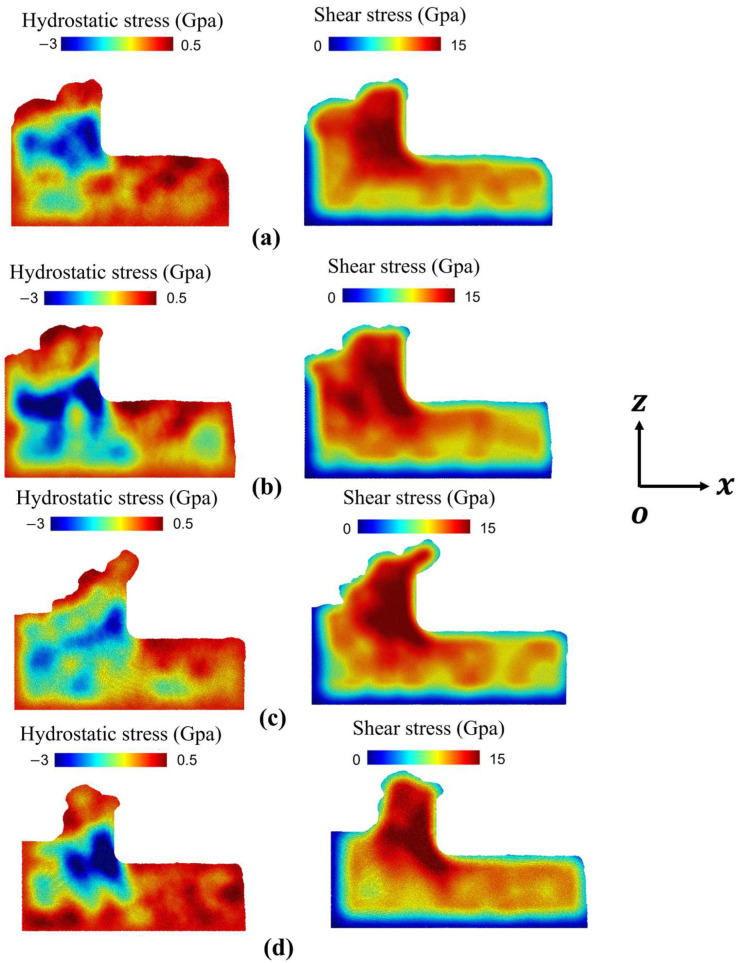
Hydrostatic stress and shear stress analysis, with grain sizes of (**a**) 25.48, (**b**) 15.83, (**c**) 12.36, and (**d**) 9.65 nm.

Figure 2 sequentially displays polycrystalline copper workpieces with grain sizes of 25.48 nm, 15.83 nm, 12.36 nm, 9.65 nm, corresponding to progressively decreasing grain sizes. In Figure 2a,b, where the grain size is relatively larger compared to that in Figure 2c,d, the cutting process involves fewer grain boundaries to hinder dislocation motion. As a result, plastic deformation within individual grains is dominated by dislocation slip, and the stacking faults generated in a single grain accumulate predominantly near the grain boundaries, leading to more pronounced stress concentration. This is reflected in the blue regions of hydrostatic stress shown in the figures. In contrast, the hydrostatic stress in Figure 2c,d is noticeably lower than in Figure 2a,b. The smaller grain size and shorter intergranular distance result in less dislocation pile-up at each grain boundary during slip, thereby reducing the severity of stress concentration.

Regarding the shear stress distribution, as the grain size decreases, dislocation nucleation and propagation within a grain encounter increases resistance from grain boundaries. A higher applied shear stress is required to activate dislocation nucleation in adjacent grains.

Notably, in Figure 2d, due to the inverse Hall–Petch effect, when the grain size decreases below 10 nm, grain deformation occurs mainly through grain boundary sliding. The grain boundaries themselves act as sources for dislocation nucleation, facilitating the activation and propagation of dislocations into neighboring grains. This leads to a tendency of reversed and more localized hydrostatic stress concentration. Consequently, due to the easier activation of dislocations in adjacent grains, the required shear stress exhibits a slight decrease.

#### 2.2.2. Analysis of Subsurface Defects in Machined Materials

**Figure 3:**
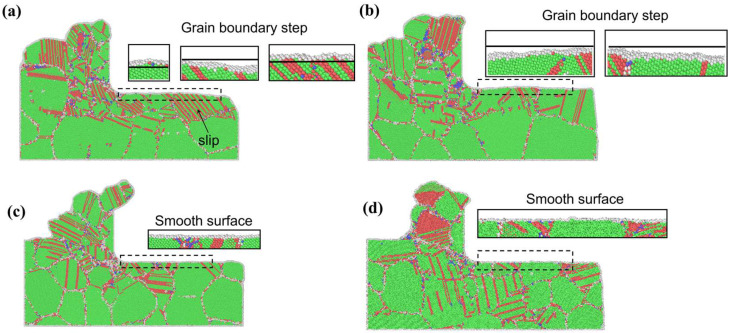
Defect analysis in workpieces with varying grain sizes of (**a**) 25.48, (**b**) 15.83, (**c**) 12.36, and (**d**) 9.65 nm.

The subsurface damage (SSD) of workpieces significantly affects their mechanical properties and surface quality, necessitating stringent control during manufacturing and usage. To analyze the subsurface damage distribution in different grains at the final simulation frame, Ovito visualization software (basic – 3.12.2) was employed. As shown in Figure 3, the extent of dislocations and stacking faults varies considerably among grains. The workpiece composed of four grain types exhibited defect concentrations primarily within the cutting layer, beneath the tool, and along grain boundary regions.

Under identical simulation conditions, a comparison between Figure 3a–d reveals that the polycrystalline copper workpiece in Figure 3a,b exhibits the generation of a large number of dislocation slips. This occurs because coarse-grained structures have larger grain boundary spacings, resulting in fewer grain boundaries traversed by the tool compared to Figure 3c,d. The reduced obstruction from grain boundaries diminishes resistance to stacking fault accumulation, leading to a higher density of stacking faults below the tool. In contrast, fine-grained materials contain a greater number of grain boundaries, which effectively impede dislocation motion during deformation, resulting in a low stacking fault density within the individual grain

Further comparison of the machined surface quality in Figure 3a–d indicates that Figure 3a,b exhibits more formation of grain boundary steps than Figure 3c,d. This difference arises because coarse grains, lacking sufficient grain boundary obstruction, allow stacking faults to accumulate more readily, promoting non-uniform plastic deformation and surface irregularities. Conversely, fine grains benefit from the Hall–Petch effect [10], where frequent dislocation blocking by grain boundaries leads to more homogeneous plastic deformation, resulting in smoother surfaces with fewer grain boundary steps compared to coarse-grained structures.

#### 2.2.3. Cutting Force

**Figure 4:**
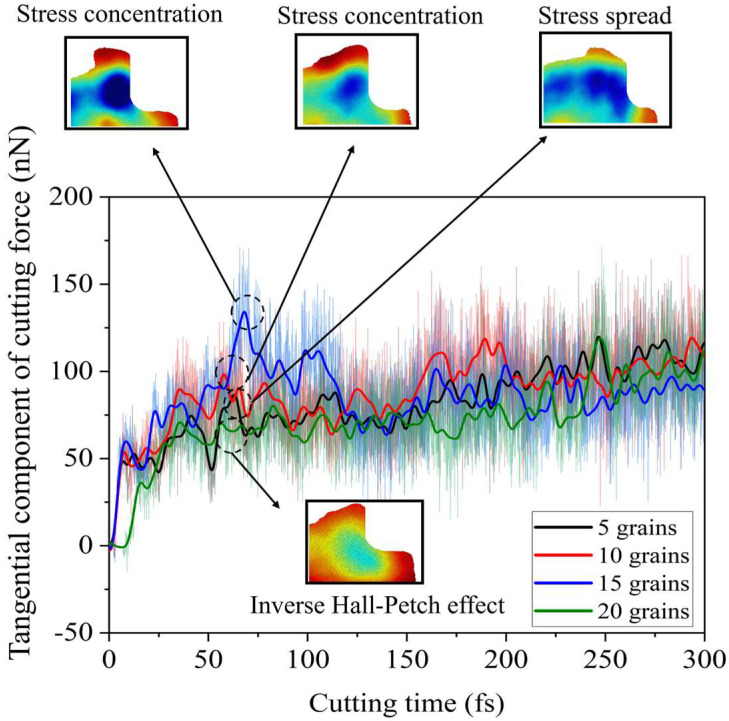
Tangential cutting force.

As shown in Figure 4, under identical simulation conditions with a cutting time of 300 fs and a cutting distance of 300 Å, the molecular dynamics (MD) simulation outputs the tangential and normal components of the cutting force. Due to the tool’s 0° rake angle, the normal component of the cutting force remains significantly smaller than the tangential component, fluctuating around zero. Since the tool begins cutting at a distance of 1 nm from the workpiece, the cutting force initially starts at zero before gradually increasing.

The tangential cutting force component reveals that the workpiece with the grain size of 12.36 nm exhibits a distinct peak at approximately 75 fs, surpassing the peak values observed in the other three grain configurations. This phenomenon occurs because the higher number of grain boundaries enhances the workpiece’s strength, increasing resistance to machining and consequently requiring greater cutting forces. Further analysis of hydrostatic stress and defects around 75 fs indicates that the tool is traversing grain boundaries at this moment, leading to significant stacking fault accumulation at these boundaries and resulting in localized stress concentration. The propagation of stress into the interior of the five-grain workpiece can be attributed to the prevalence of dislocation slip within the larger-grained material. The accumulation of a considerable number of stacking faults at grain boundaries reduces the cutting force required to activate dislocation nucleation in adjacent grains compared to fine-grained structures.

When the grain size decreases to 9.65 nm, the grain size reduces to the nanometer scale. At this point, the material’s strength or hardness no longer adheres to the conventional Hall–Petch effect but instead exhibits an inverse Hall–Petch effect [11]. This reversal causes the cutting force to decrease as the grain count increases, contrary to the trend observed in coarser-grained structures.

## 3. Experimental Investigation on the Influence of Grain Size on Machined Surface Quality

The grain size characteristics of polycrystalline copper have a significant and non-negligible influence on the material’s machinability and the resulting surface quality. During ultra-precision machining processes such as turning, the grain size and its distribution directly affect variations in cutting forces, heat generation, and chip formation, thereby impacting critical surface quality indicators including roughness and flatness [12]. Consequently, experimental evaluation of polycrystalline copper’s grain size is essential for gaining deeper insights into the material’s machining behavior and optimizing process parameters.

### 3.1. Grain Size Characterization

**Figure 5:**
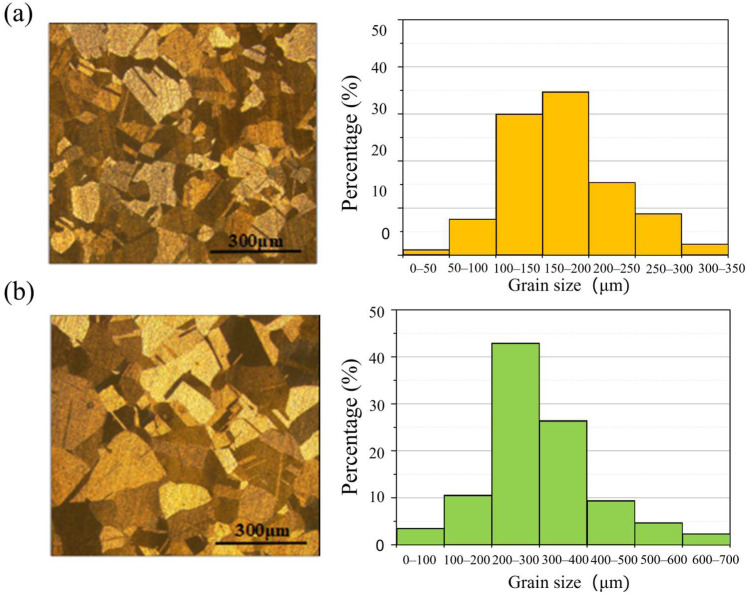
Grain size characterization with (**a**) C1020 and (**b**) C10200.

The metallographic structures of two polycrystalline copper grades (C1020 and C10200) were examined, with results presented in Figure 5. Both materials consist of equiaxed grains, with C10200 exhibiting significantly larger grain sizes compared to C1020. Quantitative analysis of grain size distribution revealed average grain diameters of 169 μm for C1020 and 300 μm for C10200. These characterized grain size parameters will serve as fundamental data for subsequent investigations into machinability and surface generation mechanisms during nanoscale cutting processes [13].

### 3.2. Experimental Design

**Figure 6:**
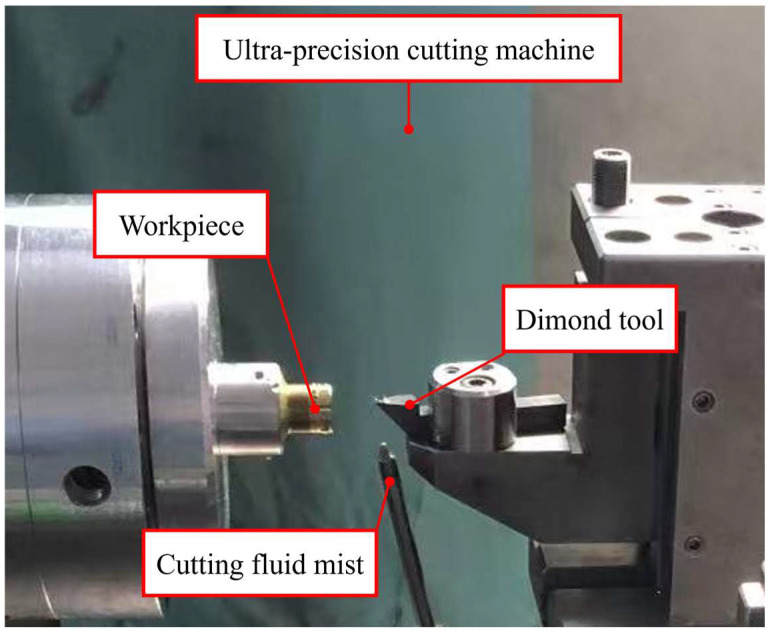
Experimental setup.

As shown in Figure 6, the experiments were conducted on an advanced horizontal ultra-precision diamond turning lathe, performing continuous rotary cutting tests to precisely observe and record the machining behavior of different grain-sized materials under controlled parameters. The cutting tests were implemented on a high-speed machining platform, where workpieces were first pre-cut with PCD tools to ensure surface parallelism with the tool path, followed by dynamic balancing adjustment. A single-crystal diamond tool was then employed for surface pre-cutting, which not only established the tool tip reference point but also removed the surface layer affected by PCD tools, guaranteeing consistent initial surface conditions for all subsequent tests.

The grain size of the polycrystalline copper served as the experimental variable in this study. Machining was performed using a diamond tool under the following conditions: a depth of cut of 5 μm, a feed rate of 5 μm/rev, and a spindle speed of 1200 rpm, with cutting parameters detailed in Table 2. The selected cutting conditions were determined based on ultra-precision turning requirements, machine tool accuracy, and dynamometer sensitivity. Excessive depth of cut or feed rate would exceed the scope of ultra-precision machining, while insufficient parameters would generate cutting forces too small for reliable dynamometer measurements. Similarly, higher cutting velocities would introduce excessive machine vibration that could compromise experimental results [14]. The established parameters thus represent an optimal balance between machining precision and measurement reliability.

### 3.3. Effect of Grain Size on Surface Quality

#### 3.3.1. Effect of Grain Size on Micro-Scale Grain Boundary Step Height

**Figure 7:**
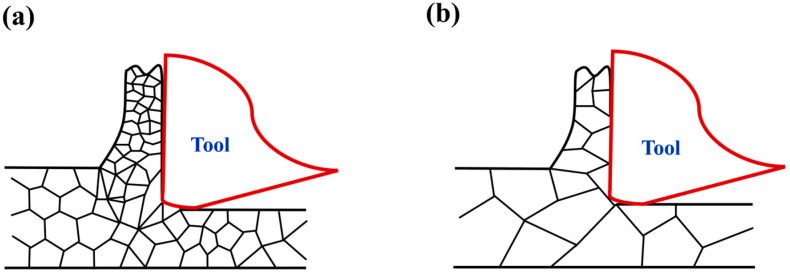
Cutting model (**a**) C1020 and (**b**) C10200.

The primary distinction between the two polycrystalline copper grades lies in their grain size characteristics, with C1020 exhibiting finer grains and consequently higher grain boundary density per unit area compared to C10200. As illustrated in Figure 7a,b, the machining process predominantly involves transgranular cutting [15] due to the tool’s exceptional sharpness and nanoscale cutting depth. Figure 7a demonstrates fine-grained material with abundant grain boundaries along the cutting path, while Figure 7b shows coarse-grained material containing significantly fewer grain boundaries during tool advancement.

**Figure 8:**
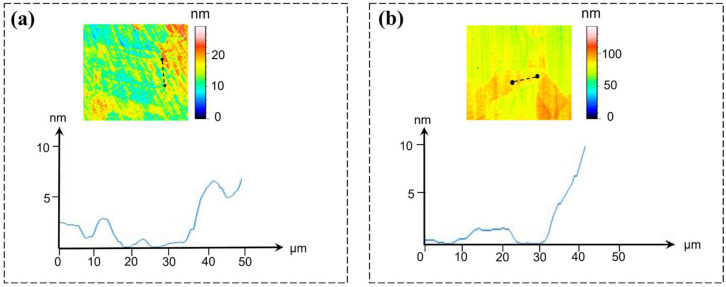
Grain boundary step height characterization with (**a**) C1020 and (**b**) C10200.

The differing mechanical properties of individual grains result in step formations at grain boundaries during tool transition between adjacent grains, a phenomenon termed grain boundary protrusion [16]. Figure 8 shows the grain boundary steps on the machined surface. For surfaces achieving nanometer-level precision, the surface roughness primarily originates from grain boundary steps of varying heights and densities formed during ultra-precision machining. The measured step height reached 20 nm under machining conditions of 5 μm depth of cut and feed per revolution. Grain boundary roughening is a contributing factor to surface roughness in ultra-precision machining [17,18,19], with surface topography analysis in Figure 8a,b revealing that C1020 specimens exhibited lower step heights than C10200, demonstrating that finer-grained materials yield superior surface finish during ultra-precision machining.

#### 3.3.2. Effect of Grain Size on Macroscopic Surface Quality

**Figure 9:**
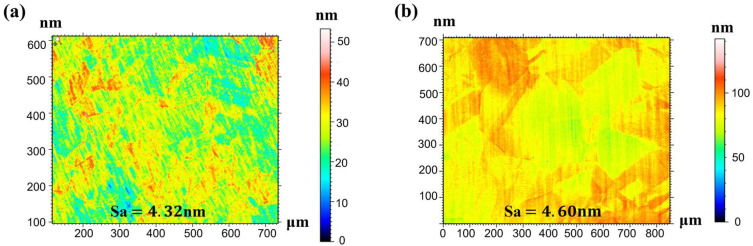
Surface roughness evaluation and comparison of (**a**) C1020 and (**b**) C10200.

To investigate grain size effects on cutting behavior, white light interferometry [20,21] was employed to characterize surface roughness parameters for both C1020 and C10200 polycrystalline coppers. The primary metric measured was the arithmetic mean height (Sa). In the study of surface topography characterization, Sa was selected as the core evaluation criterion primarily because its applicability extends from two-dimensional profile analysis to three-dimensional areal analysis, thereby enabling a more comprehensive representation of the spatial distribution characteristics of surface features. Measurements were conducted across multiple regions under varying conditions to comprehensively evaluate surface topography characteristics, with experimental results presented in Figure 9a,b.

Analysis of surface roughness distribution maps for both materials reveals significant variations in roughness parameters across different measurement regions, demonstrating inherent surface topography heterogeneity. The C10200 material exhibits elevated Sa values in specific zones, indicating rougher surfaces, while other regions show lower Sa values corresponding to smoother finishes. Similarly, the C1020 specimen displays measurable fluctuations in surface roughness parameters between measurement areas, further confirming surface non-uniformity. These observations substantiate that finer-grained materials achieve superior surface quality under identical machining conditions, manifesting both reduced roughness values and improved waviness uniformity [22].

## 4. Conclusions

This study investigates the influence of grain size on the surface quality of machined polycrystalline copper workpieces through ultra-precision cutting experiments and molecular dynamics simulations on samples with varying grain sizes. By analyzing stress distribution, subsurface defects, and cutting forces, the material removal mechanisms underlying the effect of grain size were interpreted. The main findings are as follows:

Regarding cutting forces, smaller-grained workpieces exhibit higher tangential cutting force peaks due to obstruction caused by abundant grain boundaries. This leads to an increase in cutting force with decreasing grain size. However, this trend reverses when the grain size falls below 10 nm, which is attributed to the inverse Hall–Petch effect.

In terms of surface quality, ultra-precision cutting of finer-grained copper results in more homogeneous plastic deformation and generates grain boundary steps with reduced height and density compared to larger-grained workpieces. The arithmetic mean height (Sa) value of the finished surface was measured to be 4.31 nm for the fine-grained sample, whereas the larger-grained sample exhibited a higher Sa value of 4.61 nm.

## Figures and Tables

**Table 1:** Parameters of the polycrystalline copper cutting model.

Model Parameters	Parameters
Grain Count	5, 10, 15, 20
Material	Polycrystalline copper
Material Dimensions	$500\;\times \;40\;\times \;200\;{nm}^{3}$
Timestep	1 fs
Tool Material	Diamond
Tool Edge Radius	3 nm
Tool Rake Angle	0°
Tool Clearance Angle	10°
Cutting Depth	5 nm
Cutting Velocity	0.1 nm/fs
Cutting Direction	$\left[\bar{1}\;0\;0\right]$ on (0 0 1)
Potential Function	Tersoff, Morse
Cutting Length	30 nm
Ensemble	NVT, NVE

**Table 2:** Machining parameters.

Process Parameters	Parameters
Cutting Depth	5 μm
Feed Rate	5 μm/r
Rotational Speed	1200 r/min
Coolant-Lubrication	Ethanol spray

## Data Availability

The original contributions presented in the study are included in the article, further inquiries can be directed to the corresponding author.
